# Repurposing the angiotensin II receptor blocker valsartan to inhibit penicillin-binding protein 3 and its mutants in *Haemophilus influenzae*: a comprehensive *in silico* approach

**DOI:** 10.3389/fbinf.2026.1765472

**Published:** 2026-04-28

**Authors:** Srujal Kacha, Janani Arun, Neelu Nargund, Tushar Joshi, Shalini Mathpal, Sudha Ramaiah, Anand Anbarasu

**Affiliations:** 1 Medical and Biological Computing Laboratory, School of Bioscience and Technology (SBST), Vellore Institute of Technology (VIT), Vellore, Tamil Nadu, India; 2 Department of Biotechnology, School of Bioscience and Technology (SBST), Vellore Institute of Technology (VIT), Vellore, Tamil Nadu, India; 3 Department of Biosciences, School of Bioscience and Technology (SBST), Vellore Institute of Technology (VIT), Vellore, Tamil Nadu, India

**Keywords:** drug repurposing, *Haemophilus influenzae*, molecular docking, molecular dynamic simulation, penicillin-binding protein 3, valsartan

## Abstract

**Introduction:**

Ampicillin-resistant *Haemophilus influenzae* (*H. influenzae*) has been recently designated as a medium-priority bacterial pathogen in 2024 by the World Health Organization (WHO). This pathogen is responsible for a wide range of infections, including sinusitis, acute otitis media, and pneumonia, as well as severe and life-threatening conditions such as bacteremia, meningitis, and epiglottitis. In this context, drug repurposing has emerged as an effective strategy, as the pharmacokinetic properties and safety profiles of approved drugs are already well established, allowing for faster development compared to conventional drug discovery approaches.

**Methods:**

In the current study, U.S. Food and Drug Administration (FDA)-approved drugs with structural similarity to ampicillin were filtered and evaluated using *in silico* approaches. Their pharmacokinetic properties and antimicrobial potential were assessed. Molecular docking and simulation studies were conducted to evaluate binding affinities toward wild-type penicillin-binding protein 3 (PBP_3WT_) and its common mutants (PBP3_N526K_ and PBP3_R517H_).

**Results:**

An angiotensin II receptor blocker, valsartan, demonstrated strong binding affinity toward all three target proteins, with values of −11.8 kcal/mol for PBP3_WT_, −11.4 kcal/mol for PBP3_N526K_, and −11.1 kcal/mol for PBP3_R517H_. The drug also exhibited strong intermolecular interactions and maintained stable binding with all three PBP3 variants during molecular dynamics simulations.

**Discussion:**

Based on these findings, valsartan is proposed as a potential PBP3 inhibitor targeting *H. influenzae*. The results support its candidacy for drug repurposing; however, further *in vitro* investigations are recommended to experimentally validate its antimicrobial activity.

## 1 Introduction

*Haemophilus influenzae* (*H. influenzae*) is part of the normal flora of the human respiratory tract, inhabiting the upper respiratory tract especially. This Gram-negative bacterium is an opportunistic pathogen causing a wide range of infections from sinusitis, acute otitis media, and pneumonia, to life-threatening infections, including bacteremia, meningitis, epiglottitis, and orbital cellulitis (Jordens and Slack, 1995; MacNeil et al., 2011; Jakubu et al., 2024). Annually, it causes more than 8 million cases and more than 300,000 deaths, especially in pediatric patients, worldwide (Watt et al., 2009; Nair et al., 2020). The pathogen population can be divided into two groups based on capsule formation. The capsulated *H. influenzae* population has six serotypes (a to f) based on different compositions of the capsules, while the non-capsulated isolates are also called non-typeable *H. influenzae* (NTHi) (Musser et al., 1990; Rao et al., 1999; Denizon et al., 2024). Capsulated *H. influenzae* strains occupy the upper respiratory tract of approximately 3%–5% individuals, whereas NTHi strains colonize the nasopharynx of 80% individuals (Ingham and Turk, 1969; Moxon, 1986; Slack, 2021).

According to the national surveillance data from 14 European and other countries, NTHi is responsible for 97% of non-Hib infections in invasive *H. influenzae* diseases, and a higher-than 10% mortality rate (Ladhani et al., 2010; van Wessel et al., 2011; Soeters et al., 2018; Su et al., 2023). Ampicillin is used as empirical treatment for NTHi infections currently, while broad-spectrum cephalosporins stand as alternative treatment options. However, the emergence of β-lactam resistance and multidrug resistance (MDR) has been reported as 10–25% β-lactamase-positive ampicillin-resistant (BLPAR) NTHi strains have been reported from South Africa, Europe, and America, while up to 55–57% BLPAR non-typeable *H. influenzae* strains have been documented in South Korea, Taiwan, Vietnam, and Japan (Liebowitz et al., 2003; Beekmann et al., 2005; Alpuche et al., 2007; Jansen et al., 2008; Hleba et al., 2023). The MDR strains resistant to macrolides, tetracyclines, quinolones, and trimethoprim-sulfamethoxazole are more prevalent in Asian countries than in Western countries (Gotoh et al., 2008; Niki et al., 2008; Jean et al., 2009; BAE et al., 2013). In 2024, ampicillin-resistant *H. influenzae* was included in the medium-priority group of bacterial pathogens by the World Health Organization (WHO) (WHO, 2024).

The β-lactamase enzyme produced by bacterial pathogens is the predominant defense mechanism against β-lactam antibiotics. It works by hydrolyzing them. Structural alterations in penicillin-binding proteins (PBPs) stand as another major cause of their reduced affinity toward β-lactam antibiotics, giving rise to antimicrobial resistance in the pathogen population (Jakubu et al., 2021). The PBP proteins stand as a primary target for β-lactam antibiotics, while the PBP3 variant plays an essential role in the cell division process as it contributes to septal formation during cell division, in addition to transpeptidase activity (Botta and Park, 1981; BAE et al., 2013; Kang et al., 2021; Toth et al., 2022; Sethuvel et al., 2023). In the β-lactamase-negative ampicillin-resistant (BLNAR) NTHi population, amino acid alterations near or at the canonical SXXK, SXN and KTGT/S of the PBP3 protein play a crucial role in reduced affinity toward β-lactam antibiotics (Ubukata et al., 2001; Dabernat et al., 2002; Osaki et al., 2005; Jakubu et al., 2021). Two mutations, N526K and R517H, are the most common in the PBP3 protein of *H. influenzae*. The amino acid substitution N526K is prevalent in European countries, while R517H is commonly found in bacterial isolates from Asian countries (Schotte et al., 2019; Nørskov-Lauritsen et al., 2021; Jakubu et al., 2024; Jakubu et al., 2025).

The drug repurposing approach paves the way for swift and cost-effective discovery of antibiotic alternatives for treatment. This approach represents several advantages, including less developmental time and developmental cost over traditional drug discovery, as the pre-existing pharmacokinetics and safety profiles of the drugs approved by the US Food and Drug Administration (FDA) are leveraged enabling the drug candidate to enter the Phase 2a (clinical trials) without extensive Phase 1 safety studies (Ashburn and Thor, 2004; Kepplinger, 2015; Kaul et al., 2019; Phanchana et al., 2020).

In this study, we employed a drug-repurposing approach to screen the putative penicillin-binding protein 3 inhibitor of *H. influenzae* pathogen with regard to β-lactamase resistance from the library of FDA-approved drugs with structural similarities to ampicillin. Ampicillin is the drug of choice for treating *H. influenzae* infections. It was considered a reference antibiotic due to its well-established interactions with the PBP proteins (Tipper, 1985; Raynor, 1997; Zapun et al., 2008; Kocaoglu et al., 2015; Lu et al., 2020) and to ensure the similar physicochemical properties of the FDA-approved drugs to ampicillin, which further assures a similar residual level interaction pattern and effective outer membrane penetration to access the PBP3 protein (O’Shea and Moser, 2008; Revol-Tissot et al., 2024).

The screened drug molecules were evaluated for their suitable pharmacokinetic properties and antibacterial activities, followed by assessments for their binding affinities and interaction stabilities with the wild-type PBP3 (PBP3_WT_) protein and the two prevalent PBP3 mutants, PBP3_N526K_ and PBP3_R517H._ One drug, valsartan, exhibited strong binding affinities along with strong intermolecular interactions and much more stable molecular interaction profiles with all three target proteins, compared to the reference antibiotic, ampicillin. Valsartan’s structure exhibits favorable physicochemical properties, providing efficacy against bacteria, including high polarity at physiological pH, hydrophilic nature, and increased numbers of hydrogen bond acceptor atoms, facilitating bacterial membrane penetration and improved access to the PBP3 protein.

The drug valsartan belongs to the angiotensin II receptor blocker (ARB) drug class, utilized for the treatment of hypertension, heart failure, and post-heart attack treatment. The drug prevents vasoconstriction, cellular proliferation, and cytokine and aldosterone production. Valsartan hinders angiotensin II from binding with angiotensin II receptor (type-1) (AT1), ultimately disrupting the renin angiotensin-aldosterone system (RAAS), preventing cellular actions due to angiotensin II (Miura et al., 2011; Taylor et al., 2011).

The results revealed that valsartan exhibits inhibitory potential against the wild-type PBP3 (PBP3_WT_) protein along with the two prevailing mutant variants, PBP3_R517H_ and PBP3_N526K_, in *H. influenzae*. These findings indicate that this ARB drug may disrupt the synthesis of the cell wall by targeting PBP3, underscoring its promise as a potential therapeutic candidate for managing β-lactamase-negative ampicillin-resistant (BLNAR) NTHi infections. However, *in vitro* analyses are required for further investigations.

## 2 Methodology

### 2.1 Target protein selection and structural retrieval

The penicillin-binding protein 3 (PBP 3) protein of *H. influenzae* was chosen as the target protein for the study. The Apo structure of the PBP 3 transpeptidase domain was acquired from the PDB database with the PDB ID 6HZO. The 584-amino-acid-long protein structure was obtained via the X-ray diffraction method, with 2.44 Å resolution, possessing no mutations. The protein structure was sourced in the .pdb format. Moreover, in the mutant protein structures, R517H and N526K amino acid substitutions were induced into the wild-type PBP 3 protein structure using the SPDBV tool. The conserved motifs, SXXK, responsible for transpeptidase activity of the PBP 3 protein, were considered as the active site for the site-specific molecular docking studies (Ubukata et al., 2001; Matic, 2003; Osaki et al., 2005; Papp-Wallace et al., 2012; Jakubu et al., 2025). The refined protein structures were subsequently energy minimized prior to molecular docking analysis.

### 2.2 Ligand library and structure retrieval

A library of 400 FDA-approved drugs, structurally similar to the reference drug ampicillin, was obtained from the SwissSimilarity server. The structural similarity screening was carried out using the “approved-drugs” category provided on the server. A combined method including two different molecular representations, the FP2 fingerprints (2D method) and ES5D vector (3D method), was implemented for screening, where the resulting similarity score depicts the probability of molecules sharing a common protein target. A ≥0.1 score was considered the similarity threshold (Bragina et al., 2022). The library was refined by removal of the duplicate entries and antibiotic molecules, prior to pharmacokinetics property screening. The 3rd generation cephalosporin, cefditoren (Karlowsky et al., 2002; Aguilar and Barberan, 2012), was taken as a positive control along with ampicillin. Rifampicin was used as the negative control to validate the specificity of docking and simulation protocols.

After the *in silico* screening process, the 2D SDF structures of the filtered drug molecules and the reference antibiotics were acquired from the PubChem database, followed by conversion into .pdb formats via the OpenBabel tool, prior to the following analyses.

### 2.3 Pharmacokinetic properties screening and antimicrobial activity predictions

The library of refined drug molecules was further processed through the pharmacokinetics screening. The drug molecules were filtered based on their suitability and potential therapeutic potential. The Swiss ADME server (Daina et al., 2017) was utilized to screen the drug molecules based upon basic PK/PD properties, including Lipinski’s rule violations, molecular weight, gastrointestinal (GI) absorption, bioavailability, topological polar surface area (TPSA), lipophilicity (XlogP3), and solubility (Log S). Drug molecules passing all the threshold points were taken for further screening procedures.

Antimicrobial activity predictions were carried out following ADME screening for the filtered compounds using the PASS online tool (Filimonov et al., 2014). The drug molecules predicted to have antibacterial activity were prioritized for further screening.

### 2.4 Molecular docking analysis

The pharmacokinetics property screening was followed by binding affinity analysis with the target proteins by molecular docking analysis. Binding affinity analysis of target proteins with drug molecules was carried out using AutoDock Vina software. The protein structures were optimized prior to molecular docking by performing several steps, including the addition of polar and merging of non-polar hydrogen atoms, respectively, removal of water molecules, followed by the addition of Kollman charges. In contrast, the ligand molecules were optimized by torsion fixation and addition of Gasteiger charges. Eventually, .pdbqt input files were generated for protein and ligand structures and subjected to molecular docking. The site-specific molecular docking was performed by generating a grid box with a 60 Å size and 0.375 Å equal spacing. The grid box was positioned according to the XYZ coordinates of the active site (17.382, −4.970, 11.149), obtained by PyMol. To estimate the binding affinity, 10 different conformational poses were calculated for each complex. The complex .pdb files were generated using the resulting output .pdbqt files and visualized via PyMol software.

Intermolecular interactions between the protein–ligand molecules for each docked complex were studied utilizing the LigPlot tool. The complex .pdb files were given as input files to visualize the 2D interaction plot of the respective complex.

### 2.5 Molecular dynamics simulation studies

The protein–ligand complexes with good binding affinity and strong intermolecular interactions were further evaluated for their intermolecular interaction stability via molecular dynamics simulations (MDSs). MD simulations were conducted utilizing GROMACS (GROningen MAchine for Chemical Simulation) software (2021.3 version). Protein–ligand complex files were taken as input files for MDS analysis. The CHARMM 36 force field was employed to generate the protein topologies, while ligand topologies were generated through the CGenFF server. Following topology construction, system neutralization was achieved by the addition of NaCl counter ions, using the Monte Carlo ion replacement method. Furthermore, the TIP3 model was utilized for the solvation step. System energy was minimized by performing energy minimization (1000 steps) employing the Verlet cutoff scheme and the steepest descent algorithm. Energy minimization was followed by the NVT (constant volume) and NPT (constant pressure) at 310 K temperature and 1 bar, respectively. The equilibration criteria for temperature (tau-t = 0.1 ps), pressure (tau-p = 2 ps) were set, while the energy equilibration was ensured by the minimum energy drift values. Finally, the independent MD runs were performed for a 100 ns time span for each complex, and the resulting trajectories were analyzed for convergence as well as post-simulation analyses (Mathpal et al., 2025; Priyamvada et al., 2025; Joshi et al., 2026).

The simulation results were evaluated by calculating root mean square deviation (RMSD), interaction energy (IE), root mean square fluctuation (RMSF), solvent-accessible surface area (SASA), radius of gyration (Rg), and hydrogen bonds (H-bonds) after a 100 ns MD run.

### 2.6 Binding-free energy calculations

MD simulation studies were followed by the binding-free energy (BE) calculations study, total binding-free energy, potential energy components, and free solvation energy (polar and non-polar solvation energies), including electrostatic interactions and van der Waals interactions, by implementing the molecular mechanics-generalized Born surface area (MM-GBSA) method. The final 10 ns data from the MD trajectory were used to perform the analysis.

Furthermore, the per-residue stabilizing and destabilizing complex-forming contributions were calculated for all three target protein molecules by decomposition analyses. The decomposition analysis was conducted using the gmx_MMPBSA tool.

## 3 Results

### 3.1 Active-site identification

The SXXK conserved motif present in the transpeptidase domain of the PBP3 protein was taken as the active site because these residues are mainly responsible for transpeptidase activity (Matic, 2003; Jakubu et al., 2025). The amino acid residues SXXK located at Ser327-Thr328-Val329-Lys330 were considered the active site for further *in silico* analyses.

### 3.2 Ligand library and structure retrieval

A library of 400 FDA-approved drugs, structurally similar to ampicillin, was obtained from the Swiss Similarity server. Duplicate entries and antibiotic structures were eliminated from the library, reducing the library size to 240 drugs. Following duplicates and antibiotic elimination, the pharmacokinetics screening and antimicrobial property predictions for each drug were carried out.

### 3.3 Pharmacokinetics and antimicrobial activity screening

The drug library underwent pharmacokinetic property screening by utilizing the SwissADME server. 160 drugs were retained for possessing suitable pharmacokinetic properties out of total 240 drugs, while 96 of 160 drugs were predicted for antimicrobial activities using the PASS online tool. The pharmacokinetic and antimicrobial activity profiles of the 96 screened drug molecules are provided in Supplementary Table S1.

The drug molecules were screened based upon the ideal threshold value range for each parameter, including molecular weight (150–500 g/mol), topological polar surface area (TPSA) (20–130 Å), lipophilicity (−0.7 to + 6.0), solubility (not more than 6), gastrointestinal absorption (preferably high), Lipinski’s rules (zero violations), and bioavailability score (at or more than 0.55) (Martin, 2005; Daina et al., 2017). The resulting drug, valsartan, depicted good pharmacokinetics profiles by satisfying all the considered parameters, including molecular weight (435.52 g/mol), TPSA (112.07), lipophilicity (4.39), solubility (−4.97), high gastrointestinal absorption, zero violations of Lipinski’s rules, and a 0.56 bioavailability score.

Furthermore, compared to the structural reference ampicillin, valsartan exhibits higher flexibility, with 11 rotatable bonds, than ampicillin, which has 5 rotatable bonds. The molecular weight of valsartan is slightly higher (435.52 g/mol) than that of ampicillin (349.4 g/mol).

Moreover, the drug valsartan was predicted for several potential activities using the PASS tool, including pseudolysin inhibition, muramyl tetrapeptide carboxypeptidase inhibition, and UDP-N-acetylmuramate-L-alanine ligase inhibition, indicating the drug’s antibacterial potential.

### 3.4 Molecular docking analysis

The 96 screened drugs were docked against the PBP3_WT_ protein, resulting in 13 drugs with the highest affinity with the target wild-type protein. These 13 drugs were further evaluated for their binding affinity with the mutant PBP3 proteins, PBP3_N526K_, and PBP3_R517H_ (Table 1). Among the 13, the top five drugs with good binding energy scores and intermolecular interaction profiles were further prioritized for molecular dynamics simulation (MDS) analyses to evaluate interaction stability. Of these, valsartan exhibited greater binding affinities and strong intermolecular interactions, along with stable interaction profiles in MDS analyses, with all three target proteins, than ampicillin. The lower binding energy represents higher binding affinity in the case of molecular docking (Basu et al., 2022). The binding energy of valsartan with PBP3_WT_ is −11.8 kcal/mol, which is much lower than the binding energy of the ampicillin–PBP3_WT_ complex (−6.8 kcal/mol) and the cefditoren–PBP3_WT_ complex (−8.42 kcal/mol), showcasing higher binding affinities than ampicillin and cefditoren.

**TABLE 1:** Binding energies of the screened drugs with the three target proteins.

No	Drug	BE with the PBP3_WT_ protein (kcal/mol)	BE with the PBP3_R517H_ protein (kcal/mol)	BE with the PBP3_N526K_ protein (kcal/mol)
1	Ampicillin	−6.8	−8.9	−8.9
2	Valsartan	−11.8	−11.1	−11.4
3	Sacubitril	−11.4	−10.8	−10.9
4	Moexipril	−10.6	−11	−10.5
5	Dinoprost	−10.6	−10.9	−10.7
6	Benazepril	−10.6	−10.6	−10.2
7	Carboprost	−10.1	−11.3	−10.5
8	Trandolapril	−10	−11	−9.7
9	Quinapril	−10.1	−9.4	−10.7
10	Aceclofenac	−10.5	−8.3	−10.6
11	Cilazapril	−10.1	−10.3	−9.7
12	Alvimopan	−10	−9.8	−9.9
13	Dehydrocholic acid	−10	−9.6	−10.1
14	Treprostinil	−10.3	−10	−9.3

Following molecular docking with the PBP3_WT_ protein, the drugs were docked against two mutants, PBP3_N526K_ and PBP3_R517H_. No significant change was observed in binding energy scores for the test drugs when evaluated against both mutant PBP3 variants. The drug valsartan exhibited binding energy scores of −11.1 kcal/mol with the PBP3_R517H_ protein and −11.4 kcal/mol with PBP3_R517H_. However, the reference antibiotic ampicillin showed −8.9 kcal/mol binding energy with both PBP3 mutants, while the cefditoren exhibited −7.98 kcal/mol and −8.14 kcal/mol binding for PBP3_R517H_ and PBP3_N526K_, respectively. The binding energies for all the studied complexes and the binding energies of negative control complexes are provided in Table 2.

**TABLE 2:** Molecular docking profile and intermolecular interactions of protein–ligand complexes.

Complex	Binding energy (kcal/mol)	No. of hydrogen (H) bonds	Residues involved in H-bonds	No. of hydrophobic (Hp) bonds	Residues involved in Hp bonds
Valsartan + PBP3_WT_	−11.8	2	Ser327, Ser379	12	Glu324, Lys330, Lys359, Glu360, Val362, Val364, Asn381, Tyr438, Tyr440, Thr515, Ala516, Arg517
Ampicillin + PBP3_WT_	−8.9	5	Ser327, Ser379, Asn381, Tyr438	4	Gly326, Lys330, Val364, Tyr440
Cefditoren + PBP3_WT_	−8.42	8	Ser327, Lys330, Met377, Asn381, Asn499, Arg517	10	Glu324, Gly326, Val364, Ser379, Tyr438, Tyr440, Gly514, Thr515, Ala516, Tyr528
Rifampicin + PBP3_WT_	9.92	1	Asn381	14	Ser327, Lys330, Val364, Ala365, Met377, Asn378, Ser379, Tyr438, Thr513, Gly514, Thr515, Ala516, Tyr528, Gly560
Valsartan + PBP3_R517H_	−11.1	3	Ser327, Tyr438, Tyr440	8	Gly326, Lys330, Lys359, Glu360, Val362, Asn381, Thr515, His517
Ampicillin + PBP3_R517H_	−8.9	5	Ser327, Ser379, Tyr438	5	Gly326, Lys330, Val364, Asn381, Tyr440
Cefditoren PBP3_R517H_	−7.98	5	Ser327, Lys330, Met377, Asn381, Asn499	12	Glu324, Gly326, Val364, Ser379, Tyr438, Gly439, Tyr440, Thr513, Gly514, Thr515, Ala516, His517
Rifampicin + PBP3_R517H_	6.73	1	Asn381	15	Ser327, Lys330, Val364, Ala365, Met377, Asn378, Ser379, Tyr438, Thr513, Gly514, Thr515, Ala516, His517, Tyr528, Gly560
Valsartan + PBP3_N526K_	−11.4	1	Ser327	11	Glu324, Gly326, Lys330, Lys359, Glu360, Val362, Asn381, Tyr438, Tyr440, Thr515, Arg517
Ampicillin + PBP3_N526K_	−8.9	5	Ser327, Ser379, Tyr438	5	Gly326, Lys330, Val364, Asn381, Tyr440
Cefditoren + PBP3_N526K_	−8.14	7	Ser327, Lys330, Met377, Ser379, Asn499, Asn381	9	Glu324, Gly326, Val364, Asn378, Tyr438, Gly439, Tyr440, Ala516
Rifampicin + PBP3_N526K_	6.97	1	Asn381	14	Ser327, Lys330, Val364, Ala365, Met377, Asn378, Ser379, Tyr438, Thr513, Gly514, Thr515, Ala516, Tyr528, Gly560

### 3.5 Intermolecular interactions

**FIGURE 1:**
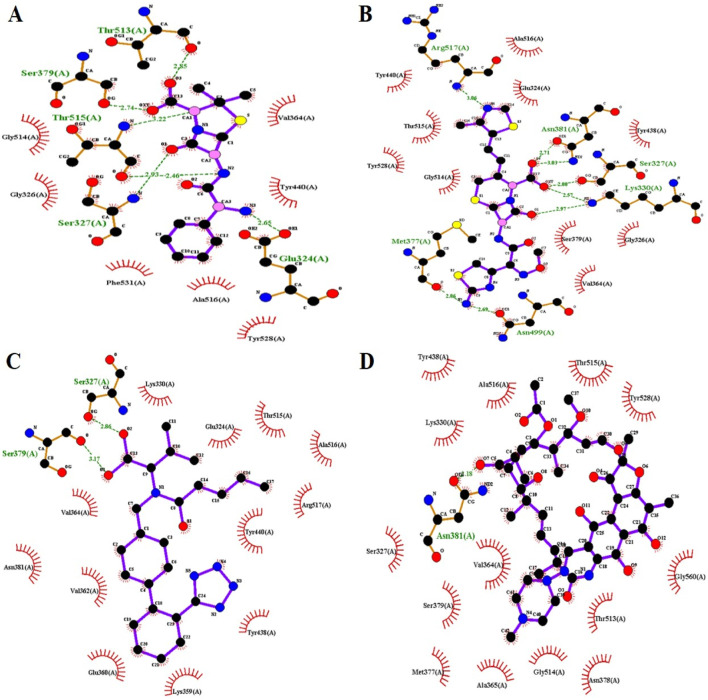
Intermolecular interactions of ampicillin **(A)**, cefditoren **(B)**, valsartan **(C)**, and rifampicin **(D)** with the PBP3_WT_ protein.

**FIGURE 2:**
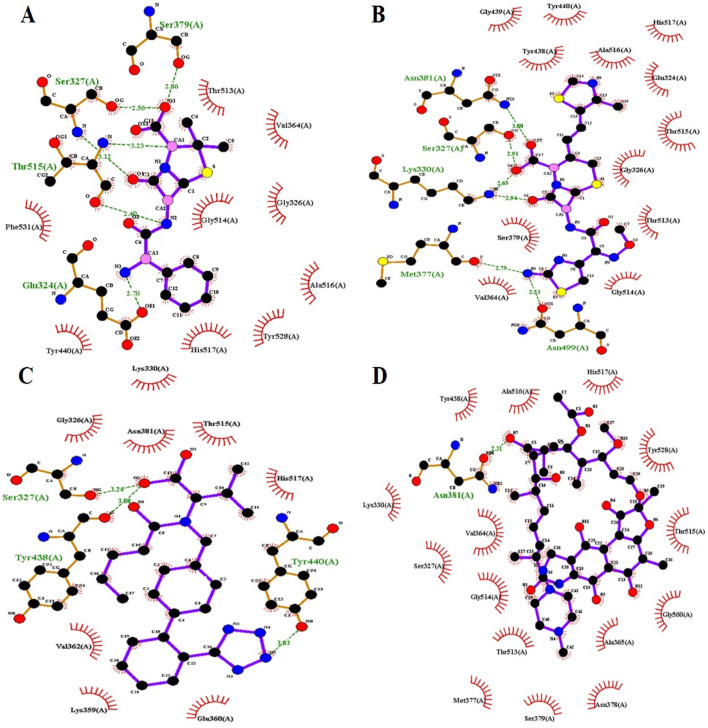
Intermolecular interactions of ampicillin **(A)**, cefditoren **(B)**, valsartan **(C)**, and rifampicin **(D)** with the PBP3_R517H_ protein.

**FIGURE 3:**
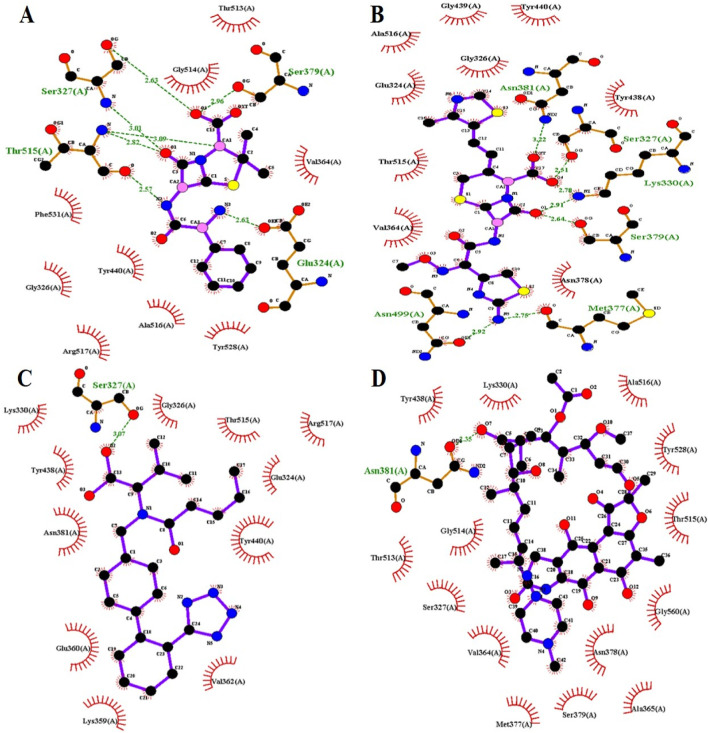
Intermolecular interactions of ampicillin **(A)**, cefditoren **(B)**, valsartan **(C)**, and rifampicin **(D)** with the PBP3_N526K_ protein.

**FIGURE 4:**
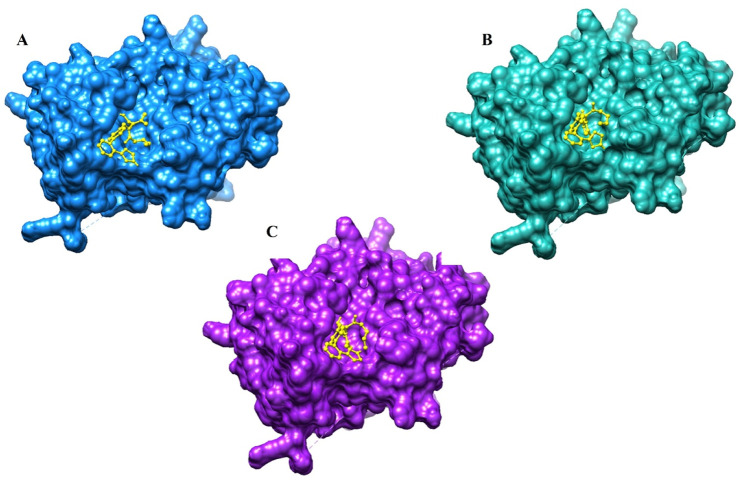
3D interaction of valsartan with the PBP3_WT_ protein **(A)**, with the PBP3_R517H_ protein **(B)**, and with the PBP3_N526K_ protein **(C)**.

Valsartan established strong intermolecular interactions with all three target PBP3 proteins by forming hydrogen (H) and hydrophobic (Hp) bonds with active-site amino acids and surrounding residues. Ampicillin formed five hydrogen bonds and four hydrophobic bonds with the PBP3_WT_ protein, while cefditoren formed 8 hydrogen bonds and 10 hydrophobic bonds (Figures 1A,B). Moreover, ampicillin formed five hydrogen and five hydrophobic bonds with both PBP3 mutant proteins, PBP3_N526K_ and PBP3_R517H_ (Figures 2A, 3A), while cefditoren formed 5 hydrogen and 12 hydrophobic bonds with PBP3_R517H_ and 7 hydrogen and 9 hydrophobic bonds with PBP3_N526K_ (Figures 2B, 3B). In comparison, valsartan demonstrated strong binding to all three PBP3 variants. It maintained stable interactions with the PBP3_WT_ protein (Figure 1C). Valsartan interacted with the PBP3_R517H_ mutant by forming three hydrogen bonds and eight hydrophobic interactions involving the key active-site residues Ser327 and Lys330 (Figure 2C). Likewise, 1 hydrogen bond (Ser327) and 11 hydrophobic interactions were made with the PBP3_N526K_ mutant, including those with Lys330 (Figure 3C). The intermolecular interactions of rifampicin (negative control) with PBP3_WT_, PBP3_R517H_, and PBP3_N526K_ are provided in Figures 1D, 2D, 3D, respectively. The 3D interaction poses of valsartan with all three PBP3 proteins are depicted in Figure 4.

Molecular docking and intermolecular interaction studies of the established β-lactams ampicillin and cefditoren against the PBP3 protein provided an additional advantage of active-site (SXXK) validation, as their intermolecular interactions involve hydrogen bond formation with active-site residues Ser327 and Lys330 and hydrophobic bond formation with surrounding residues. Valsartan also exhibits a similar interaction pattern, depicting the strong binding affinity with the active site.

Together, these interactions highlight valsartan’s potential to effectively inhibit both wild-type and mutant forms of PBP3. The detailed intermolecular interactions between protein and ligand molecules for each complex are shown in Table 2.

### 3.6 Molecular dynamics simulations (MDS)

The protein–ligand complexes with good molecular docking profiles were further assessed for their interaction stabilities by performing molecular dynamics simulations for 100 ns. Based on the RMSD, RMSF, IE, Rg, SASA, and H-bond calculation results, the drugs were analyzed for their interaction stability in complex with the PBP3 wild-type and mutant proteins. Valsartan emerged as the best drug with greater interaction stability profiles during MDS analyses, compared with the reference antibiotic ampicillin, cefditoren, and the negative control, rifampicin (Table 3).

**TABLE 3:** Stability and flexibility profiles of protein–ligand complexes obtained by molecular dynamics simulations.

Protein–ligand complex	RMSD (nm)	RMSF (nm)	IE (kJ/mol)	Rg (nm)	SASA (nm^2^)	H-bonds
Valsartan + PBP3_WT_	0.24 ± 0.017	0.10 ± 0.05	−133.05 ± 18.59	1.86 ± 0.009	133.36 ± 2.65	3
Ampicillin + PBP3_WT_	3.68 ± 1.14	0.09 ± 0.05	−17.55 ± 0.24	1.88 ± 0.014	137.01 ± 3.22	2
Cefditoren + PBP3_WT_	1.89 ± 0.43	0.10 ± 0.04	−248 ± 21.69	1.86 ± 0.008	132 ± 2.21	8
Rifampicin + PBP3_WT_	2.92 ± 1.40	0.10 ± 0.05	−117.62 ± 16.04	1.87 ± 0.007	131.07 ± 1.97	3
Valsartan + PBP3_R517H_	0.21 ± 0.013	0.10 ± 0.049	−167.86 ± 17.60	1.86 ± 0.0084	134. 06 ± 2.02	3
Ampicillin + PBP3_R517H_	2.92 ± 1.45	0.10 ± 0.05	−57.02 ± −9.89	1.86 ± 0.008	134.22 ± 2.22	2
Cefditoren PBP3_R517H_	2.55 ± 1.15	0.11 ± 0.05	−112.29 ± 0.79	1.86 ± 0.007	134 ± 2.03	4
Rifampicin + PBP3_R517H_	2.97 ± 1.58	0.10 ± 0.048	−81.34 ± −14.45	1.85 ± 0.008	133.07 ± 1.87	2
Valsartan + PBP3_N526K_	0.25 ± 0.034	0.11 ± 0.076	−102.18 ± 0.14	1.87 ± 0.009	138.07 ± 3.21	3
Ampicillin + PBP3_N526K_	1.12 ± 1.36	0.11 ± 0.05	−80.83 ± 6.42	1.88 ± 0.01	136.21 ± 2.37	3
Cefditoren + PBP3_N526K_	3.62 ± 0.84	0.11 ± 0.07	−78.71 ± 2.42	1.89 ± 0.01	139.44 ± 2.73	4
Rifampicin + PBP3_N526K_	2.02 ± 0.65	0.11 ± 0.05	−132 ± 23.62	1.86 ± 0.009	133.82 ± 2.53	4

#### 3.6.1 Root mean square deviation (RMSD)

**FIGURE 5:**
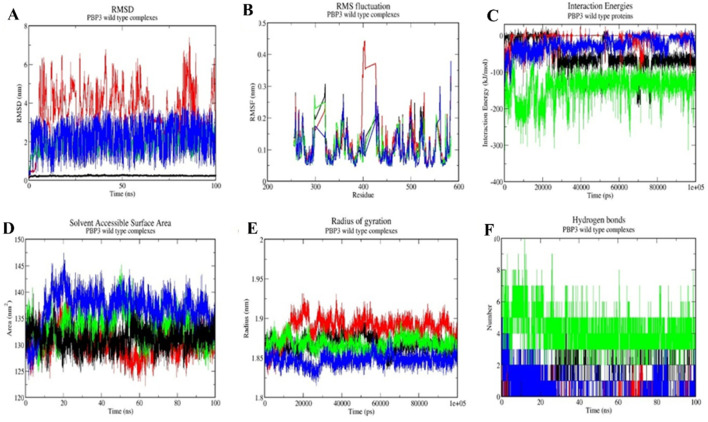
Molecular dynamics simulation profiles of ampicillin (red), valsartan (black), cefditoren (blue), and rifampicin (green) with the PBP3_WT_ protein. **(A)** RMSD as a function of time, **(B)** RMSF across residues, **(C)** interaction energy profiles, **(D)** radius of gyration, **(E)** solvent accessible surface area, and **(F)** hydrogen bond count over the simulation period.

**FIGURE 6:**
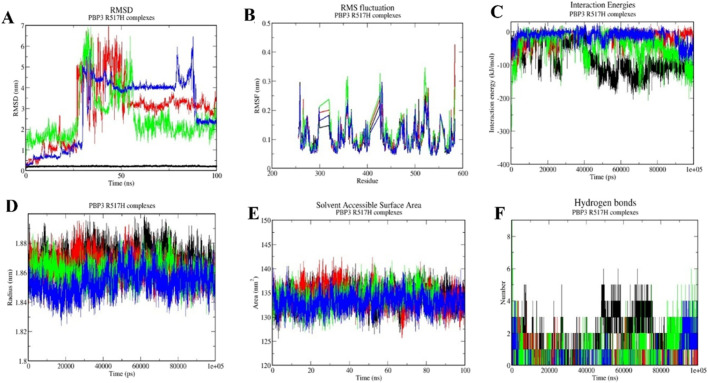
Molecular dynamics simulation profiles of ampicillin (red), valsartan (black), cefditoren (blue), and rifampicin (green) with the PBP3_R517H_ protein. **(A)** RMSD as a function of time, **(B)** RMSF across residues, **(C)** interaction energy profiles, **(D)** radius of gyration, **(E)** solvent accessible surface area, and **(F)** hydrogen bond count over the simulation period.

**FIGURE 7:**
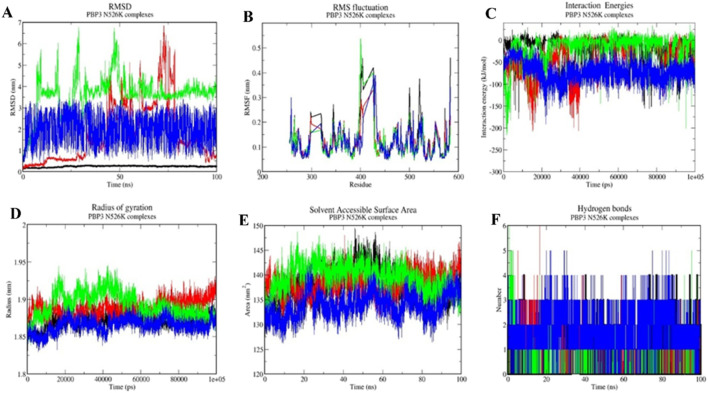
Molecular dynamics simulation profiles of ampicillin (red), valsartan (black), cefditoren (blue), and rifampicin (green) with the PBP3_N526K_ protein. **(A)** RMSD as a function of time, **(B)** RMSF across residues, **(C)** interaction energy profiles, **(D)** radius of gyration, **(E)** solvent accessible surface area, and **(F)** hydrogen bond count over the simulation period.

Root mean square deviation (RMSD) analysis helps evaluate the conformational shifts and overall stability of a protein–ligand complex during the MD simulation time frame (100 ns). The RMSD value for valsartan with the PBP3_WT_ protein was 0.24 ± 0.017 nm, while the RMSD values of the mutant PBP3_R517H_ and PBP3_N526K_ proteins are 0.21 ± 0.013 nm and 0.25 ± 0.034 nm, respectively. However, the RMSD values for ampicillin complexes were 3.68 ± 1.14 nm, 2.92 ± 1.45 nm, and 1.12 ± 1.36 nm. The RMSD values for cefditoren were 1.89 ± 0.43 nm, 2.55 ± 1.15 nm, and 3.62 ± 0.84 nm for PBP3_WT_, PBP3_R517H_, and PBP3_N526K_ complexes, respectively (Figures 5A, 6A, 7A) and (Table 3).

#### 3.6.2 Root mean square fluctuation (RMSF)

Root mean square fluctuation (RMSF) analyses portray fluctuations at the protein residual level during MD simulation in complex with a ligand molecule. The RMSF values for ampicillin complexes were 0.09 ± 0.05 nm, 0.10 ± 0.05 nm, and 0.11 ± 0.05 nm for the PBP3_WT,_ PBP3_R517H_, and PBP3_N526K_ protein complexes, respectively. RMSF values for cefditoren complexes with the PBP3_WT,_ PBP3_R517H_, and PBP3_N526K_ proteins were 0.10 ± 0.04 nm, 0.11 ± 0.05 nm, and 0.11 ± 0.07 nm, respectively. RMSF values for valsartan were 0.10 ± 0.05 nm, 0.10 ± 0.049 nm, and 0.10 ± 0.05 nm for the PBP3_WT,_ PBP3_R517H_, and PBP3_N526K_ protein complexes, respectively (Figures 5B, 6B, 7B) and (Table 3).

#### 3.6.3 Interaction energy (IE)

Interaction energy (IE) represents the energy consumed by protein–ligand molecules for intermolecular interaction during the molecular dynamics simulation. The interaction energies for valsartan were −133.05 ± 18.59 kJ/mol, −167.86 ± 17.60 kJ/mol, and −102.18 ± 0.14 kJ/mol, respectively, for the PBP3_WT,_ PBP3_R517H_, and PBP3_N526K_ protein complexes. However, ampicillin showed higher interaction energies than valsartan, −17.55 ± 0.24 kJ/mol with PBP3_WT_, −57.02 ± −9.89 kJ/mol with PBP3_R517H_, and −80.83 ± 6.42 kJ/mol in complex with PBP3_N526K_. The IE values for cefditoren were −248 ± 21.69 kJ/mol with PBP3_WT_, −112.29 ± 0.79 kJ/mol with PBP3_R517H_, and −78.2.42 kJ/mol with PBP3_N526K_ (Figures 5C, 6C, 7C; Table 3).

#### 3.6.4 Radius of gyration (Rg)

The radius of gyration assesses protein–ligand complex stability by calculating structural compactness utilizing the 100 ns trajectory file of the studied complex. The Rg values of valsartan were 1.86 ± 0.009 nm, 1.86 ± 0.0084 nm, and 1.87 ± 0.009 nm for the PBP3_WT,_ PBP3_R517H_, and PBP3_N526K_ protein complexes, respectively. However, the ampicillin complexes resulted in 1.88 ± 0.014 nm (PBP3_WT_), 1.86 ± 0.008 nm (PBP3_R517H_), and 1.88 ± 0.01 nm (PBP3_N526K_) Rg values. The cefditoren complexes exhibited 1.86 ± 0.008 nm, 1.86 ± 0.007 nm, and 1.89 ± 0.01 nm Rg values for the PBP3_WT_, PBP3_R517H_, and PBP3_N526K_ protein complexes (Figures 5D, 6D, 7D) and (Table 3).

#### 3.6.5 Solvent accessible surface area (SASA)

Solvent accessible surface area (SASA) analysis is carried out to evaluate the total accessible surface area of the protein by solvent (water), throughout the MD simulation time frame. SASA values for ampicillin complexes were 137.01 ± 3.22 nm^2^ (PBP3_WT_), 134.22 ± 2.22 nm^2^ (PBP3_R517H_), and 136.21 ± 2.37 nm^2^ (PBP3_N526K_). The SASA values for cefditoren complexes for the PBP3_WT_, PBP3_R517H_, and PBP3_N526K_ proteins were 132 ± 2.21 nm^2^, 134 ± 2.03 nm^2^ and 139 ± 2.73 nm^2^, respectively. The SASA values for valsartan drug complexes were 133.36 ± 2.65 nm^2^, 134. 06 ± 2.02 nm^2^, and 138.07 ± 3.21 nm^2^ for the PBP3_WT,_ PBP3_R517H_, and PBP3_N526K_ proteins, respectively (Figures 5E, 6E, 7E) and (Table 3).

#### 3.6.6 Hydrogen bond (H-bond)

The hydrogen bonding pattern between protein and ligand molecules during MD simulation time for each complex was analyzed by using the 100 ns simulation trajectory files. Valsartan formed three hydrogen bonds with all three target proteins during 100 ns MD simulations. Ampicillin formed two H-bonds with the PBP3_WT_ and PBP3_R517H_ proteins and three H-bonds with the PBP3_N526K_ protein. Cefditoren formed eight, four, and four hydrogen bonds with PBP3_WT,_ PBP3_R517H_, and PBP3_N526K_, respectively (Figures 5F, 6F, 7F) and (Table 3).

Overall, when all the post-MD simulation analyses were considered, the drug valsartan establishes more stable interactions with all three target proteins than ampicillin, cefditoren, and rifampicin.

### 3.7 Molecular mechanics/generalized Born surface area (MMGBSA) analysis

**FIGURE 8:**
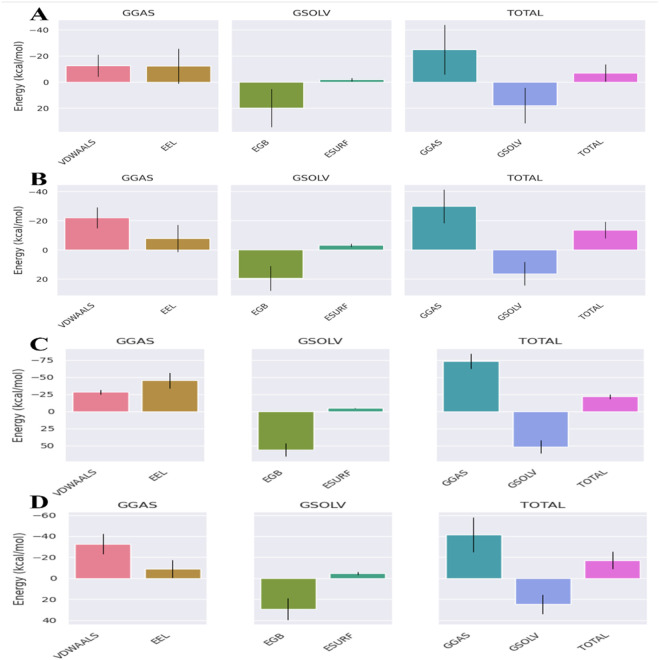
MMGBSA binding-free energy contribution for **(A)** PBP3_WT_ + ampicillin, **(B)** PBP3_WT_ + valsartan, **(C)** PBP3_WT_ + cefditoren, and **(D)** PBP3_WT_ + rifampicin.

**FIGURE 9:**
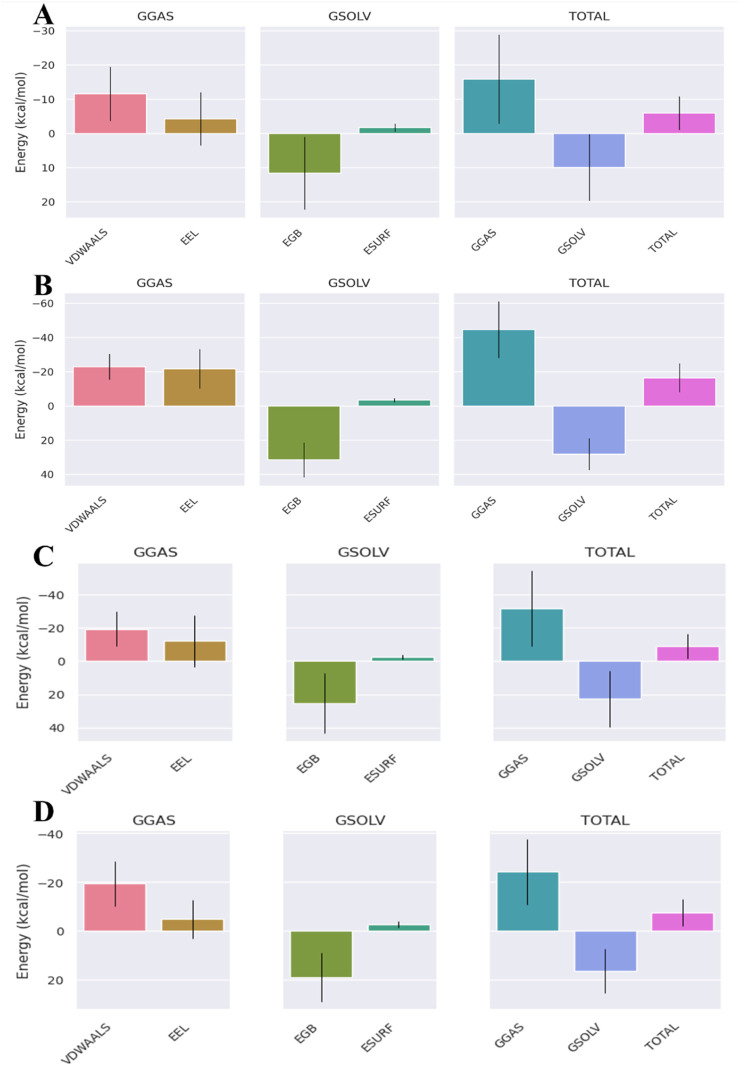
MMGBSA binding-free energy contributions for **(A)** PBP3_R517H_ + ampicillin, **(B)** PBP3_R517H_ + valsartan, **(C)** PBP3_R517H_ + cefditoren, and **(D)** PBP3_R517H_ + rifampicin.

**FIGURE 10:**
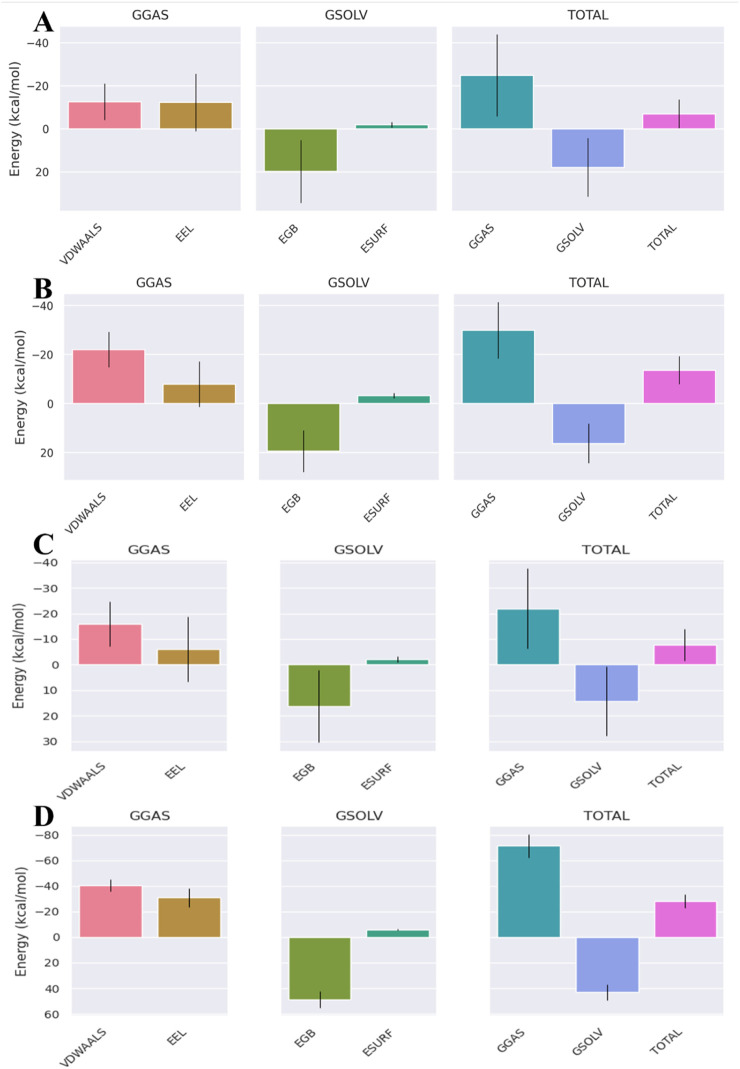
MMGBSA binding-free energy contributions for **(A)** PBP3_N526K_ + ampicillin, **(B)** PBP3_N526K_ + valsartan, **(C)** PBP3_N526K_ + cefditoren and **(D)** PBP3_N526K_ + rifampicin.

The binding-free energies for the ampicillin complexes were much higher than those of the valsartan complexes, suggesting the efficient binding of valsartan with the target proteins. The binding-free energies of the valsartan complexes were −16.25 kcal/mol, −13.56 kcal/mol, and −16.39 kcal/mol for the PBP3_WT_, PBP3_N526K_, and PBP3_R517H_ protein complexes, respectively. However, for the ampicillin complexes, the binding-free energies for the PBP3_WT_, PBP3_N526K_, and PBP3_R517H_ protein complexes were −1.13 kcal/mol, −6.94 kcal/mol, and −5.92 kcal/mol, respectively (Figures 8–10). Among the different energy components, van der Waals interactions contribute predominantly to the total binding-free energy, exhibiting the highest negative values of up to −22 kcal/mol commonly for all three valsartan complexes and up to −12 kcal/mol for the ampicillin complexes, followed by electrostatic and solvation energies. Conversely, the positive values of the Generalized Born electrostatic solvation energies (EGBs) signify an unfavorable polar solvation contribution. The binding-free energies of all complexes are provided in Table 4.

**TABLE 4:** MMGBSA profiles of the studied wild-type and mutant protein–ligand complexes.

Protein– ligand complex	Vdwaals (kcal/mol)	EEL (kcal/mol)	EGB (kcal/mol)	ESURF (kcal/mol)	GGAS (kcal/mol)	GSOLV (kcal/mol)	TOTAL (kcal/mol)
Valsartan + PBP3_WT_	−21.33	−16.79	25.14	−3.27	−38.12	21.87	−16.25
Ampicillin + PBP3_WT_	−3.12	−2.55	5	−0.47	−5.67	4.54	−1.13
Cefditoren + PBP3_WT_	−28.33	−45.15	56.32	−4.57	−73.48	51.75	−21.73
Rifampicin + PBP3_WT_	−32.6	−9.03	29.26	−4.57	−41.63	24.69	−16.94
Valsartan + PBP3_N526K_	−21.98	−7.9	19.46	−3.15	−29.88	16.31	−13.56
Ampicillin + PBP3_N526K_	−12.59	−12.23	19.8	−1.92	−24.82	17.88	−6.94
Cefditoren + PBP3_N526K_	−15.9	−6.07	16.3	−2.07	−21.97	14.23	−7.74
Rifampicin + PBP3_N526K_	−40.39	−30.87	48.77	−5.63	−71.27	43.13	−28.13
Ampicillin + PBP3_R517H_	−11.58	−4.27	11.63	−1.69	−15.86	9.94	−5.92
Valsartan + PBP3_R517H_	−22.86	−21.67	31.52	−3.38	−44.53	28.14	−16.39
Cefditoren + PBP3_R517H_	−19.47	−12.24	25.39	−2.7	−31.71	22.69	−9.02
Rifampicin + PBP3_R517H_	−19.25	−4.83	19.02	−2.47	−24.08	16.54	−7.53

### 3.8 Decomposition analysis

**FIGURE 11:**
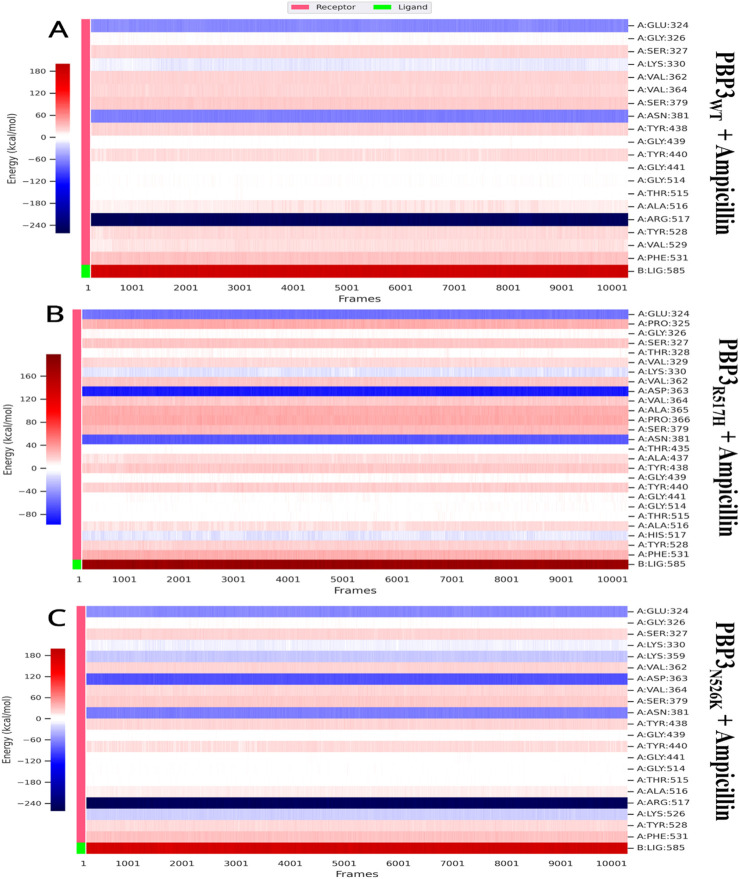
Per-residue binding energy contribution obtained via decomposition analysis: **(A)** PBP3_WT_ + ampicillin, **(B)** PBP3_R517H_ + ampicillin, and **(C)** PBP3_N526K_ + ampicillin.

**FIGURE 12:**
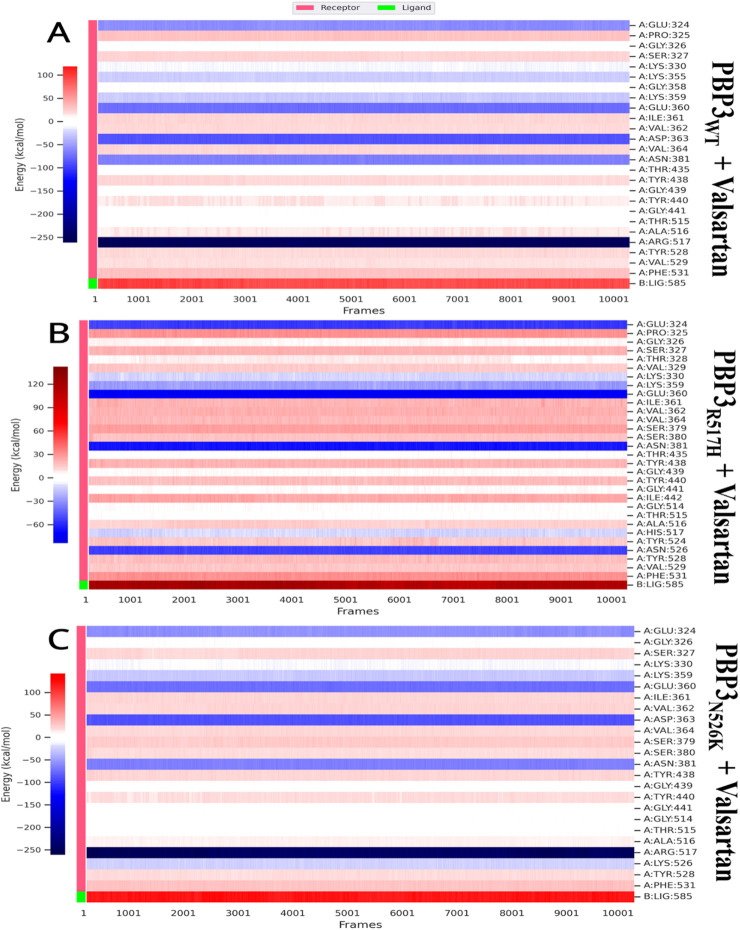
Per-residue binding energy contribution obtained via decomposition analysis: **(A)** PBP3_WT_ + valsartan, **(B)** PBP3_R517H_ + valsartan, and **(C)** PBP3_N526K_ + valsartan.

The decomposition analysis was performed to evaluate the per-residue contribution for complex establishment, as the residues may exert stabilizing or destabilizing effects over complex formation, depending on their respective binding energy contributions. The protein residues GLU324 and ASN381 were found to be common protein residues, contributing positively to each protein–ligand complex. The protein residue ARG517 contributes positively to PBP3_WT_ and PBP3_N526K_ complex formation with valsartan and ampicillin, with higher energies (Table 5) and (Figures 11, 12).

**TABLE 5:** Decomposition analysis results for per-residue binding energy contribution.

Ampicillin complex						Valsartan complex					
PBP3_WT_ residue	Average BE contribution (kcal/mol)	PBP_N526K_ residue	Average BE contribution (kcal/mol)	PBP3_R517H_ residue	Average BE contribution (kcal/mol)	PBP3_WT_ residue	Average BE contribution (kcal/mol)	PBP_N526K_ residue	Average BE contribution (kcal/mol)	PBP3_R517H_ residue	Average BE contribution (kcal/mol)
GLU:324	−60.76	GLU:324	−60.55	GLU:324	−54.27	GLU:324	−58.47	GLU:324	−56.4	GLU:324	−54.88
GLY:326	6.42	GLY:326	5.88	PRO:325	31.85	PRO:325	29.93	GLY:326	6.67	PRO:325	30.78
SER:327	22.12	SER:327	21.8	GLY:326	5.63	GLY:326	5.94	SER:327	21.47	GLY:326	6.05
LYS:330	−11.49	LYS:330	−11.31	SER:327	22.39	SER:327	21.88	LYS:330	−9.64	SER:327	21.98
VAL:362	21.98	LYS:359	−28.73	THR:328	5.19	LYS:330	−10.97	LYS:359	−28.07	THR:328	5.54
VAL:364	20.68	VAL:362	21.7	VAL:329	14.74	LYS:355	−25.74	GLU:360	−74.39	VAL:329	14.64
SER:379	26.67	ASP:363	−88.24	LYS:330	−10.83	GLY:358	6.79	ILE:361	20.73	LYS:330	−11.32
ASN:381	−65.95	VAL:364	20.16	VAL:362	22.2	LYS:359	−27.83	VAL:362	21.42	LYS:359	−27.07
TYR:438	21.13	SER:379	26.61	ASP:363	−87.85	GLU:360	−74.96	ASP:363	−88.66	GLU:360	−74.21
GLY:439	4.23	ASN:381	−66.16	VAL:364	20.71	ILE:361	20.48	VAL:364	19.99	ILE:361	21.22
TYR:440	17.9	TYR:438	20.94	ALA:365	31.77	VAL:362	19.14	SER:379	26.52	VAL:362	22.2
GLY:441	3.47	GLY:439	4.43	PRO:366	33	ASP:363	−88.33	SER:380	17.31	VAL:364	20.17
GLY:514	5.08	TYR:440	17.42	SER:379	26.67	VAL:364	19.92	ASN:381	−65.7	SER:379	26.57
THR:515	2.3	GLY:441	3.5	ASN:381	−64.43	ASN:381	−65.92	TYR:438	20.89	SER:380	16.95
ALA:516	14.55	GLY:514	4.47	THR:435	3.4	THR:435	2.15	GLY:439	3.94	ASN:381	−65.29
ARG:517	−248.89	THR:515	3.21	ALA:437	13.7	TYR:438	19.2	TYR:440	17.77	THR:435	2.95
TYR:528	18.88	ALA:516	12.89	TYR:438	21.02	GLY:439	4.42	GLY:441	3.55	TYR:438	21.11
VAL:529	15.75	ARG:517	−249.39	GLY:439	5.17	TYR:440	15.76	GLY:514	4.33	GLY:439	4.96
PHE:531	30.93	LYS:526	−24.96	TYR:440	18.97	GLY:441	3.62	THR:515	1.79	TYR:440	18.63
		TYR:528	19.08	GLY:441	4.32	THR:515	1.65	ALA:516	12.54	GLY:441	4.52
		PHE:531	31.31	GLY:514	4.3	ALA:516	15.55	ARG:517	−246.01	ILE:442	24.48
				THR:515	2.78	ARG:517	−248.85	LYS:526	−23.57	GLY:514	4.45
				ALA:516	13.62	TYR:528	18.86	TYR:528	17.69	THR:515	3.05
				HIS:517	−10.65	VAL:529	15.51	PHE:531	30.57	ALA:516	13.35
				TYR:528	17.87	PHE:531	30.4			HIS:517	−11.3
				PHE:531	31					TYR:524	15.11
										ASN:526	−52.85
										TYR:528	18.58
										VAL:529	15.53

## 4 Discussion

*Haemophilus influenzae* (*H. influenzae*) can cause a diverse array of infections, from simple otitis media and epiglottitis to fatal infections like meningitis and pneumonia, especially in pediatric and immunocompromised populations (Tristram et al., 2007; Nair et al., 2020). Bacterial diseases caused by the BLNAR NT *H. influenzae* are generally treated by targeting PBP3 proteins using β-lactam antibiotics like penicillin, cephalosporins, and their combinations (Abavisani et al., 2024). In the present era of antimicrobial resistance, the high antibiotic resistance in *H. influenzae* is increasing, due to mutations in target proteins, loss of porins, horizontal gene transfers, and production of β-lactamase enzymes, resulting in the inefficiency of even last-line antibiotic treatment options (Lobb et al., 2023; Jakubu et al., 2025).

PBP3 serves as a critical target in BLNAR non-typeable *Haemophilus influenzae* due to its essential role in septal ring formation during cell division and in peptidoglycan synthesis. Among multidrug-resistant isolates, two PBP3 mutants, PBP3_N526K_ and PBP3_R517H_, are the most frequently observed, predominantly in European and Asian MDR strains, respectively. In a study by Jakubu et al. (2024), isolates carrying the N526K mutation exhibited up to an eightfold increase in MIC values for ampicillin and cefuroxime. These mutations are responsible for the disruption of a critical and conserved hydrogen bond, including three amino acids, Arg517, Asn526, and Glu324, stabilizing the loop surrounding the catalytic site (SXXK) of PBP3. The mutations result in the rearrangement of the active pocket lining and decreased hydrogen bond interactions necessary for SXXK–β-lactam binding, eventually providing resistance against β-lactam antibiotics (Ubukata et al., 2001; Osaki et al., 2005; Bellini et al., 2019; Reist et al., 2019; Denizon et al., 2024). These findings highlight the significant impact of these amino acid substitutions in driving antibiotic resistance in BLNAR NT *H. influenzae* populations (BAE et al., 2013; Nørskov-Lauritsen et al., 2021; Toth et al., 2022; Jakubu et al., 2024).

Drug repurposing approach has emerged as the most convenient, time-effective, and cost-effective drug discovery approach to combat antimicrobial resistance at the present time. Due to the known pharmacodynamics, safety profiles, and dosage regimens, the drug development process becomes more feasible, providing opportunities to identify the antimicrobial activities of FDA-approved drugs rapidly and repurpose them, coping with AMR more efficiently. Some classic examples of drug repurposing include auranofin, an anti-rheumatoid arthritis drug, identified for broad-spectrum antimicrobial activity against and currently under clinical trials for the treatment of gastrointestinal protozoa (Debnath et al., 2012; Kaul et al., 2019). The drug auranofin has also been found to possess antibacterial activity against *Clostridium difficile*, *Staphylococcus aureus* (*S. aureus*), and vancomycin-resistant *enterococci* (VRE) (Thangamani et al., 2016; AbdelKhalek et al., 2019; Kang et al., 2021). An anti-allergic drug, chlorcyclizine, which was identified for antiviral activity, and antitumor agents, such as eflornithine and miltefosine, have been repurposed for the treatment of human African trypanosomiasis and visceral leishmaniasis (Smorenburg et al., 2000; SIMARRO et al., 2012; He et al., 2015; Kaul et al., 2019). The NSAIDs ketorolac and etodolac have been identified for their ability to cross the blood–brain barrier and show PBP inhibition potential against *Streptococcus pneumoniae* (Basu et al., 2022). Benzydamine has been reported to enhance the antimicrobial efficacy of doxycycline in combating methicillin-resistant *S. aureus*, VRE, and uropathogenic *Escherichia coli* (UPEC) (Liu et al., 2021). Similarly, docetaxel has been identified as an inhibitor of the histidinol phosphate aminotransferase (HisC) enzyme in UPEC, resulting in suppressed bacterial growth (Kaur et al., 2022). Several drugs, including amenamevir, duvelisib, nilotinib, and lifitegrast, have been presented as potential therapeutics against *Salmonella typhi* by dihydrofolate reductase (DHFR) inhibition (Joshi et al., 2022). Other drugs identified and repurposed for their antibacterial activities include niclosamide (an anthelminthic drug), ivacaftor (an anticystic fibrosis drug), DPIC (a nitric oxide synthase inhibitor), pentamidine (an antiprotozoal drug), and disulfiram (an anti-alcohol drug) (Imperi et al., 2013; Xu et al., 2016; Singh et al., 2017; Thakare et al., 2017; Thakare et al., 2019; Das et al., 2019; Kaul et al., 2019).

Several structurally similar scaffolds, including cephalosporin analogs and flavonoids, have also been reported recently for their effectiveness against the mutant bacterial target proteins. Joshi et al. (2026) reported five flavonoid compounds, theaflavin, neobavaisoflavone, trifolirhizin, isosilybinin, and glycitin, containing structural similarities with the known GyrB inhibitors novobiocin, chlorobiocin, and coumermycin A1 as potential inhibitors against the wild-type GyrB as well as mutant GyrB (E466D) from *K. pneumoniae* (Joshi et al., 2026). Two ciprofloxacin analogs, C1 and C5, exhibited notable inhibition potential against the GyrA_WT_ and three GyrA mutants (S83F, D87G, and D87N) from fluoroquinolone-resistant *Salmonella typhi* (Guchhait and Ramaiah, 2025). Similarly, N-benzylquinoline-8-sulfonamide, a quinolone analog, has been presented as a promising *P. aeruginosa* GyrA inhibitor effective against wild-type and mutant GyrA proteins (Ghosh and Ramaiah, 2025).

In our study, we employ a drug repurposing approach to evaluate FDA-approved drugs against *H. influenzae* by targeting the PBP3 protein. The active site for PBP3 protein and two mutants, PBP3_N526K_ and PBP3_R517H_ was identified as Ser(327)-Thr(328)-Val(329)-Lys(330). Ampicillin was set as the lead molecule and used as a reference drug for this study. The drugs were screened for the drug repurposing approach from the ligand library, based on their pharmacokinetic properties, antimicrobial activities, and binding affinity toward target proteins, and further analyses were performed. The selected drug molecules were first subjected to molecular docking against both the wild-type and mutant PBP3 proteins, followed by MD simulations, MMGBSA, and decomposition analyses. Among the screened compounds, valsartan, which is an angiotensin II receptor blocker, demonstrated a markedly higher binding affinity, stable intermolecular interactions, and lower binding-free energies against all three target proteins. Valsartan yielded a docking score of −11.8 kcal/mol, which is substantially lower than that of the reference antibiotic ampicillin (−8.9 kcal/mol), when docked against PBP3_WT_. The higher binding affinity from molecular docking does not explicitly confirm the antibacterial activity. MD simulations were carried out for further validations. The MDS results confirm the formation of more stable complexes by valsartan with the target proteins then ampicillin as valsartan-PBP3_WT_ complex exhibited lower RMSD values (0.24 ± 0.017 nm) and interaction energy values (−133.05 ± 18.59 kJ/mol), while the ampicillin–PBP3_WT_ complex resulted in higher RMSD and IE values of 3.68 ± 1.14 nm and −17.55 ± 0.24 kJ/mol, respectively, indicating unstable complex formation. A similar pattern was observed when the drug was evaluated against both PBP3 mutants. Valsartan represented significant binding energies and established stable complexes with PBP3_R517H_ and PBP3_N526K_ compared to ampicillin complexes. Valsartan demonstrated a strong docking score of −11.1 kcal/mol (PBP3_R517H_) and −11.4 kcal/mol (PBP3_N526K_), along with the lower RMSD values, 0.21 ± 0.013 nm and 0.25 ± 0.034 nm, and IE values, −167.86 ± 17.60 kJ/mol with PBP3_R517H_ and −102.18 ± 0.14 kJ/mol with PBP3_N526K._

The MMGBSA analyses revealed much lower binding free energy scores for the valsartan complexes than for the ampicillin complexes. Based on the overall results, valsartan showed greater stability and stronger binding with the wild-type and mutant PBP3 proteins than ampicillin, highlighting its potential as a computationally predicted PBP3 inhibitor.

Valsartan, marketed under the brand name Diovan, is an FDA-approved angiotensin II receptor blocker (ARB) used for hypertension (high blood pressure), heart failure (in patients intolerant of ACE inhibitors), and some renal-protection indications (e.g., diabetic nephropathy) (de Gasparo and Whitebread, 1995; McInnes, 1999). As it is a selective antagonist of the angiotensin II type 1 (AT_1_) receptor, it blocks angiotensin II, which mediates vasoconstriction and releases aldosterone (Markham and Goa, 1997). It also supports vasodilation and reduces blood pressure and cardiac afterload (de Gasparo and Whitebread, 1995). Prior research has highlighted the antibacterial activity of valsartan. Ohra et al. (2024) confirmed its inhibitory effect on the peptidoglycan deacetylase (SpPgdA) enzyme in *Streptococcus pneumoniae*. The drug has also been investigated *in vitro* and *in vivo* for potential repurposing in the treatment of diabetic foot ulcers (El-Salamouni et al., 2021; Paul et al., 2022; Ohra et al., 2024). The drug has been reported for its repositioning potential for Alzheimer’s disease treatment as valsartan is capable of lowering brain β-amyloid protein levels, ultimately benefiting Aβ-related memory deficits (Wang et al., 2007).

The ARB drug valsartan exhibits higher polarity (TPSA = 122.07 Å^2^), six hydrogen bond acceptors, and two hydrogen bond donors, along with the logD_7.4_ value of 2.478. The high polarity at physiological pH and increased numbers of hydrogen bond acceptors promote favorable interactions of valsartan with the outer membrane proteins, facilitating passive diffusion towards periplasmic PBP3 through the outer membrane. The logD_7.4_ value 2.478 depicts the hydrophilic nature of valsartan, indicating an advantageous feature for Gram-negative (*H. influenzae*) membrane penetration specifically (Brown et al., 2014). These physiochemical properties collectively align with the ideal physiochemical properties of Gram-negative antibacterials, known to facilitate their passage through the outer membrane porins and access periplasmic PBP3 protein, with enhanced intracellular accumulation due to reduced susceptibility to efflux proteins, preventing their extraction by the bacterial cell body (O’Shea and Moser, 2008; Brown et al., 2014; Revol-Tissot et al., 2024). Hence, based on these physicochemical properties, valsartan may effectively inhibit the PBP3 protein from *H. influenzae*.

Finally, we propose that the FDA-approved drug valsartan is a potential inhibitor of PBP3 wild-type and PBP3_R517H_ and PBP3_N526K_ proteins, based on the depiction of its strong and stable binding energies and intermolecular interactions with all three target PBP3 proteins from *H. influenzae*. *In vitro* experiments are required to confirm the *in silico* results and assess valsartan’s PBP3 inhibition potential under biological conditions. Nevertheless, the *in silico* study provides a robust preliminary computational framework, highlighting the ARB drug, valsartan, as a potential antibacterial candidate for drug repurposing against BLNAR *H. influenzae* infections and providing the rational basis for subsequent *in vitro* assessments.

## 5 Conclusion

Ampicillin-resistant *H. influenzae* remains a major concern due to increasing antibiotic resistance and high mortality and morbidity rates. The drug repurposing approach stands as a promising and conventional approach to tackle the current AMR situation. The study reveals the antibacterial potential of the ARB drug valsartan against the *H. influenzae* pathogen by inhibiting wild-type PBP3 and the two prevalent mutants PBP3_R517H_ and PBP3_N526K_. Findings from the comprehensive *in silico* workflow involving thorough pharmacokinetic and antimicrobial activity screening, molecular docking, MD simulations, MMGBSA, and decomposition analyses confirm the PBP3 inhibitory efficiency of the ARB drug valsartan. The drug also exhibits strong binding affinity and forms stable complexes with both prominent PBP3 mutants, indicating its potential relevance against the MDR *H. influenzae* isolates. However, the experimental validations are crucial prior to consideration of valsartan as a therapeutic option for BLNAR NT *H. influenzae* infections.

## Data Availability

The original contributions presented in the study are included in the article/Supplementary Material, further inquiries can be directed to the corresponding author.
