# Supplementary material for: Repurposing the angiotensin II receptor blocker valsartan to inhibit penicillin-binding protein 3 and its mutants in Haemophilus influenzae: a comprehensive in silico approach
Source: Front Bioinform. 2026 Apr 28;6:1765472. doi: 10.3389/fbinf.2026.1765472 (PMC13160910; doi:10.3389/fbinf.2026.1765472)
Supplement: Supplementary file 1 [file Table1.docx]

**Supplementary File 1** **–** Pharmacokinetics and Antimicrobial activity profiles of the screened drug molecules

| **Molecule** | **CID** | **MW** | **TPSA** | **XLOGP3** | **ESOL Log S** | **GI absorption** | **Lipinski #violations** | **Bioavailability Score** | **PASS Activity** | **Level** | **Pa** | **Pi** |
| --- | --- | --- | --- | --- | --- | --- | --- | --- | --- | --- | --- | --- |
| Aceclofenac | 71771 | 354.18 | 75.63 | 4.32 | -4.68 | High | 0 | 0.56 | Antiviral | Pa>Pi | 0,388 | 0,104 |
| Acemetacin | 1981 | 415.82 | 94.83 | 4.19 | -4.91 | High | 0 | 0.56 | Antiviral | Pa>0,3 | 0,400 | 0,090 |
| Albuterol Sulfate | 39859 | 239.31 | 72.72 | 0.31 | -1.45 | High | 0 | 0.55 | Cell wall biosynthesis inhibitor | Pa> Pi | 0,095 | 0,069 |
| Alclofenac | 30951 | 226.66 | 46.53 | 2.48 | -2.77 | High | 0 | 0.85 | Cell wall biosynthesis inhibitor | Pa> Pi | 0,094 | 0,073 |
| Alvimopan | 5488548 | 424.53 | 89.87 | 1.68 | -3.22 | High | 0 | 0.55 | Antiviral | Pa>Pi | 0,213 | 0,184 |
| Ambrisentan | 6918493 | 378.42 | 81.54 | 3.8 | -4.59 | High | 0 | 0.56 | Antiviral | Pa>0,3 | 0,402 | 0,089 |
| Amlexanox | 2161 | 298.29 | 106.42 | 3.08 | -3.97 | High | 0 | 0.56 | Antibacterial | Pa>0,3 | 0,390 | 0,032 |
| Artesunate | 6917864 | 384.42 | 100.52 | 1.88 | -3.08 | High | 0 | 0.56 | Antiviral | Pa>0,3 | 0,334 | 0,187 |
| Ataluren | 11219835 | 284.24 | 76.22 | 3.09 | -3.95 | High | 0 | 0.56 | Antibacterial | Pa>0,3 | 0,205 | 0,113 |
| Bempedoic Acid | 10472693 | 344.49 | 94.83 | 4.77 | -4.06 | High | 0 | 0.56 | Antibacterial | Pa>Pi | 0,248 | 0,084 |
| Benazepril | 5362124 | 424.49 | 95.94 | 1.26 | -2.89 | High | 0 | 0.55 | Antiviral | Pa>0,3 | 0,483 | 0,058 |
| Bendamustine Hydrochloride | 77082 | 358.26 | 58.36 | 2.89 | -3.58 | High | 0 | 0.85 | Antiviral | Pa>0,7 | 0,574 | 0,016 |
| Benoxaprofen | 39941 | 301.72 | 63.33 | 3.23 | -4.08 | High | 0 | 0.85 | Cell wall biosynthesis inhibitor | Pa> Pi | 0,241 | 0,078 |
| Bentiromide | 6957673 | 404.42 | 115.73 | 3.26 | -4.25 | High | 0 | 0.56 | Antibacterial | Pa>Pi | 0,161 | 0,155 |
| Bepotastine | 164522 | 388.89 | 62.66 | 1.03 | -2.7 | High | 0 | 0.55 | Antiviral | Pa>0,3 | 0,372 | 0,126 |
| Bezafibrate | 39042 | 361.82 | 75.63 | 3.8 | -4.3 | High | 0 | 0.56 | Anti-Helicobacter pylori | Pa>0,3 | 0,202 | 0,125 |
| Biotin | 171548 | 244.31 | 103.73 | 0.33 | -1.23 | High | 0 | 0.56 | Antibacterial | Pa>0,3 | 0,357 | 0,041 |
| Bromfenac | 60726 | 334.16 | 80.39 | 3.29 | -4.16 | High | 0 | 0.56 | Cell wall biosynthesis inhibitor | Pa> Pi | 0,197 | 0,167 |
| Bumetanide | 2471 | 364.42 | 127.1 | 4.54 | -4.79 | High | 0 | 0.56 | Antibacterial | Pa>Pi | 0,131 | 0,096 |
| Captopril | 44093 | 217.29 | 96.41 | 0.34 | -1.14 | High | 0 | 0.56 | Antibacterial | Pa>Pi | 0,144 | 0,068 |
| Carboprost | 5281075 | 368.51 | 97.99 | 2.91 | -3.17 | High | 0 | 0.56 | Antibacterial | Pa>0,3 | 0,337 | 0,047 |
| Carprofen | 2581 | 273.71 | 53.09 | 4.05 | -4.46 | High | 0 | 0.85 | Cell wall biosynthesis inhibitor | Pa>0,3 | 0,279 | 0,037 |
| Cerivastatin Sodium | 23663992 | 459.55 | 99.88 | 3.58 | -4.49 | High | 0 | 0.56 | Antiviral | Pa>0,3 | 0,484 | 0,031 |
| Cetirizine Hydrochloride | 55182 | 388.89 | 53.01 | 1.7 | -3.12 | High | 0 | 0.55 | Cell wall biosynthesis inhibitor | Pa> Pi | 0,126 | 0,026 |
| Chlorambucil | 2708 | 304.21 | 40.54 | 1.7 | -2.44 | High | 0 | 0.85 | Antiviral | Pa>0,7 | 0,699 | 0,010 |
| Cilazapril | 56330 | 417.5 | 99.18 | 0.55 | -2.33 | High | 0 | 0.55 | Cell wall biosynthesis inhibitor | Pa> Pi | 0,116 | 0,036 |
| Ciprofibrate | 2763 | 289.15 | 46.53 | 3.42 | -3.77 | High | 0 | 0.85 | Antibacterial | Pa>0,3 | 0,303 | 0,059 |
| Clometacin | 33176 | 357.79 | 68.53 | 4.04 | -4.72 | High | 0 | 0.85 | Anti-Helicobacter pylori | Pa>0,3 | 0,215 | 0,104 |
| Clorazepic Acid | 2809 | 314.72 | 78.76 | 3.28 | -4.13 | High | 0 | 0.56 | Cell wall biosynthesis inhibitor | Pa>0,3 | 0,461 | 0,003 |
| Dapagliflozin Propanediol | 24906252 | 408.87 | 99.38 | 2.35 | -3.78 | High | 0 | 0.55 | Antibacterial | Pa>Pi | 0,261 | 0,077 |
| Deferasirox | 214348 | 373.36 | 108.47 | 3.8 | -4.89 | High | 0 | 0.56 | Antibacterial | Pa>Pi | 0,204 | 0,113 |
| Dehydrocholic acid | 6674 | 402.52 | 88.51 | 2.56 | -3.68 | High | 0 | 0.56 | Antibacterial | Pa>Pi | 0,281 | 0,067 |
| Dexibuprofen | 39912 | 206.28 | 37.3 | 3.5 | -3.36 | High | 0 | 0.85 | Antibacterial | Pa>Pi | 0,214 | 0,105 |
| Dexketoprofen | 667550 | 254.28 | 54.37 | 3.12 | -3.59 | High | 0 | 0.85 | Antibacterial | Pa>Pi | 0,183 | 0,134 |
| Diclofenac sodium | 5018304 | 296.15 | 49.33 | 4.4 | -4.65 | High | 0 | 0.85 | Cell wall biosynthesis inhibitor | Pa>0,3 | 0,222 | 0,110 |
| Difenoxin | 34328 | 424.53 | 64.33 | 2.71 | -4.13 | High | 0 | 0.55 | Antiviral | Pa>0,3 | 0,302 | 0,225 |
| Dinoprost | 5280363 | 354.48 | 97.99 | 2.72 | -2.96 | High | 0 | 0.56 | Antibacterial | Pa>0,3 | 0,379 | 0,035 |
| Empagliflozin | 11949646 | 450.91 | 108.61 | 2.03 | -3.8 | High | 0 | 0.55 | Antibacterial | Pa>Pi | 0,201 | 0,117 |
| Enalapril maleate | 5388961 | 376.45 | 95.94 | -0.07 | -1.57 | High | 0 | 0.55 | Antiviral | Pa>0,3 | 0,552 | 0,012 |
| Ethacrynic acid | 3278 | 303.14 | 63.6 | 3.82 | -3.96 | High | 0 | 0.85 | Antiviral | Pa>0,7 | 0,516 | 0,020 |
| Febuxostat | 134018 | 316.37 | 111.45 | 3.9 | -4.3 | High | 0 | 0.56 | Antiviral | Pa>0,3 | 0,350 | 0,159 |
| Fenbufen | 3335 | 254.28 | 54.37 | 3.2 | -3.57 | High | 0 | 0.85 | Antibacterial | Pa>0,3 | 0,202 | 0,115 |
| Fenclofenac | 65394 | 297.13 | 46.53 | 4.8 | -4.91 | High | 0 | 0.85 | Anti-Helicobacter pylori | Pa>0,3 | 0,227 | 0,086 |
| Fenclozic acid | 28858 | 253.7 | 78.43 | 2.74 | -3.45 | High | 0 | 0.56 | Antibacterial | Pa>Pi | 0,183 | 0,134 |
| Fenofibrate | 3339 | 318.75 | 63.6 | 3.91 | -4.35 | High | 0 | 0.85 | Cell wall biosynthesis inhibitor | Pa>0,3 | 0,284 | 0,034 |
| Fenoprofen | 3342 | 242.27 | 46.53 | 3.31 | -3.66 | High | 0 | 0.85 | Antibacterial | Pa>Pi | 0,196 | 0,121 |
| Flurbiprofen Sodium | 23684814 | 244.26 | 37.3 | 4.16 | -4.27 | High | 0 | 0.85 | Antibacterial | Pa>0,3 | 0,218 | 0,102 |
| Fospropofol Disodium | 3038497 | 288.28 | 85.8 | 2.46 | -3.01 | High | 0 | 0.56 | Cell wall biosynthesis inhibitor | Pa> Pi | 0,095 | 0,070 |
| Gabapentin Enacarbil | 9883933 | 329.39 | 101.93 | 3.13 | -3.19 | High | 0 | 0.56 | Antibacterial | Pa>0,3 | 0,300 | 0,060 |
| gamma Linolenic acid | 5280933 | 278.43 | 37.3 | 4.77 | -4.06 | High | 0 | 0.56 | Antibacterial | Pa>0,3 | 0,335 | 0,047 |
| Gemfibrozil | 3463 | 250.33 | 46.53 | 3.76 | -3.61 | High | 0 | 0.85 | Cell wall biosynthesis inhibitor | Pa>0,3 | 0,200 | 0,158 |
| Glafenine | 3474 | 372.8 | 91.68 | 3.46 | -4.32 | High | 0 | 0.55 | Antiviral | Pa>0,3 | 0,628 | 0,005 |
| Guaifenesin | 3516 | 198.22 | 58.92 | 1.39 | -1.93 | High | 0 | 0.55 | Antibacterial | Pa>Pi | 0,207 | 0,111 |
| Hydroxyzine | 3658 | 374.9 | 35.94 | 3.7 | -4.31 | High | 0 | 0.55 | Cell wall biosynthesis inhibitor | Pa>Pi | 0,099 | 0,062 |
| Ibufenac | 15250 | 192.25 | 37.3 | 3.35 | -3.2 | High | 0 | 0.85 | Antibacterial | Pa>Pi | 0,207 | 0,111 |
| Ibuprofen sodium dihydrate | 23690315 | 206.28 | 37.3 | 3.5 | -3.36 | High | 0 | 0.85 | Antibacterial | Pa>Pi | 0,214 | 0,105 |
| Imipramine Pamoate | 24904 | 280.41 | 6.48 | 4.8 | -4.76 | High | 0 | 0.55 | Antibacterial | Pa>Pi | 0,128 | 0,105 |
| Indomethacin | 3715 | 357.79 | 68.53 | 4.27 | -4.86 | High | 0 | 0.85 | Cell wall biosynthesis inhibitor | Pa>0,3 | 0,193 | 0,178 |
| Indoprofen | 3718 | 281.31 | 57.61 | 2.77 | -3.55 | High | 0 | 0.85 | Antiviral | Pa>0,7 | 0,456 | 0,045 |
| Ketorolac Tromethamine | 84003 | 255.27 | 59.3 | 2.72 | -3.37 | High | 0 | 0.85 | Antibacterial | Pa>Pi | 0,154 | 0,051 |
| Lesinurad | 53465279 | 404.28 | 93.31 | 4.71 | -5.45 | High | 0 | 0.56 | Antiviral | Pa>0,7 | 0,769 | 0,004 |
| Levalbuterol | 123600 | 239.31 | 72.72 | 0.31 | -1.45 | High | 0 | 0.55 | Cell wall biosynthesis inhibitor | Pa> Pi | 0,095 | 0,069 |
| Levocetirizine | 1549000 | 388.89 | 53.01 | 1.7 | -3.12 | High | 0 | 0.55 | Cell wall biosynthesis inhibitor | Pa> Pi | 0,126 | 0,026 |
| Lumiracoxib | 151166 | 293.72 | 49.33 | 4.16 | -4.46 | High | 0 | 0.85 | Antiviral | Pa>0,3 | 0,330 | 0,194 |
| Mephenesin | 4059 | 182.22 | 49.69 | 1.41 | -1.94 | High | 0 | 0.55 | Antibacterial | Pa>Pi | 0,207 | 0,111 |
| Moexipril | 91270 | 498.57 | 114.4 | 1.18 | -3.06 | High | 0 | 0.55 | Antiviral | Pa>0,3 | 0,525 | 0,017 |
| Mycophenolate Sodium | 23665584 | 320.34 | 93.06 | 3.2 | -3.64 | High | 0 | 0.56 | Antibacterial | Pa>0,3 | 0,446 | 0,022 |
| Naproxen Sodium | 23681059 | 230.26 | 46.53 | 3.34 | -3.61 | High | 0 | 0.85 | Antibacterial | Pa>Pi | 0,170 | 0,146 |
| Nedocromil | 50294 | 371.34 | 126.81 | 2.22 | -3.59 | High | 0 | 0.56 | Antibacterial | Pa>0,3 | 0,404 | 0,029 |
| Olopatadine | 5281071 | 337.41 | 49.77 | 1.47 | -2.88 | High | 0 | 0.55 | Cell wall biosynthesis inhibitor | Pa> Pi | 0,195 | 0,173 |
| Oxaprozin Potassium | 23694685 | 293.32 | 63.33 | 4.19 | -4.54 | High | 0 | 0.85 | Cell wall biosynthesis inhibitor | Pa>0,3 | 0,235 | 0,087 |
| Perindopril Erbumine | 441313 | 368.47 | 95.94 | 0.91 | -2.04 | High | 0 | 0.55 | Antiviral | Pa>0,3 | 0,535 | 0,015 |
| Pioglitazone | 4829 | 356.44 | 93.59 | 3.75 | -4.31 | High | 0 | 0.55 | Antiviral | Pa>0,3 | 0,351 | 0,157 |
| Pirbuterol hydrochloride | 68658 | 240.3 | 85.61 | -0.06 | -1.22 | High | 0 | 0.55 | Antibacterial | Pa>Pi | 0,159 | 0,156 |
| Piretanide | 4849 | 362.4 | 118.31 | 3.92 | -4.58 | High | 0 | 0.56 | Antibacterial | Pa>Pi | 0,162 | 0,041 |
| Pirprofen | 35935 | 251.71 | 40.54 | 2.94 | -3.32 | High | 0 | 0.85 | Antibacterial | Pa>Pi | 0,129 | 0,102 |
| Potassium canrenoate | 23671691 | 358.47 | 74.6 | 1.9 | -3.06 | High | 0 | 0.85 | Antiviral | Pa>0,3 | 0,370 | 0,129 |
| Pravastatin Sodium | 16759173 | 424.53 | 124.29 | 2.49 | -3.31 | High | 0 | 0.56 | Antibacterial | Pa>0,3 | 0,419 | 0,026 |
| Probenecid | 4911 | 285.36 | 83.06 | 3.21 | -3.4 | High | 0 | 0.56 | Antibacterial | Pa>Pi | 0,175 | 0,142 |
| Proglumide | 4922 | 334.41 | 86.71 | 2.4 | -2.82 | High | 0 | 0.56 | Antiviral | Pa>0,3 | 0,425 | 0,026 |
| Quinapril | 54892 | 438.52 | 95.94 | 1.24 | -2.89 | High | 0 | 0.55 | Antiviral | Pa>0,3 | 0,545 | 0,013 |
| Ramipril | 5362129 | 416.51 | 95.94 | 1.43 | -2.75 | High | 0 | 0.55 | Antiviral | Pa>0,3 | 0,500 | 0,024 |
| Rosiglitazone | 77999 | 357.43 | 96.83 | 3.11 | -3.91 | High | 0 | 0.55 | Antiviral | Pa>Pi | 0,290 | 0,098 |
| Sacubitril | 9811834 | 411.49 | 92.7 | 3.74 | -4.19 | High | 0 | 0.56 | Antiviral | Pa>0,3 | 0,485 | 0,030 |
| Suprofen | 5359 | 260.31 | 82.61 | 3.31 | -3.73 | High | 0 | 0.56 | Cell wall biosynthesis inhibitor | Pa> Pi | 0,243 | 0,075 |
| Tafamidis | 11001318 | 308.12 | 63.33 | 4.21 | -4.83 | High | 0 | 0.85 | Antibacterial | Pa>0,3 | 0,225 | 0,098 |
| Tiagabine Hydrochloride | 91274 | 375.55 | 97.02 | 2.65 | -3.74 | High | 0 | 0.55 | Antiviral | Pa>0,3 | 0,357 | 0,148 |
| Tiaprofenic acid | 5468 | 260.31 | 82.61 | 3.35 | -3.75 | High | 0 | 0.56 | Cell wall biosynthesis inhibitor | Pa> Pi | 0,195 | 0,171 |
| Tienilic acid | 38409 | 331.17 | 91.84 | 4.1 | -4.55 | High | 0 | 0.56 | Cell wall biosynthesis inhibitor | Pa> Pi | 0,235 | 0,087 |
| Tolmetin | 5509 | 257.28 | 59.3 | 2.79 | -3.36 | High | 0 | 0.85 | Cell wall biosynthesis inhibitor | Pa> Pi | 0,194 | 0,175 |
| Trandolapril | 5484727 | 430.54 | 95.94 | 1.97 | -3.17 | High | 0 | 0.55 | Antiviral | Pa>0,3 | 0,500 | 0,024 |
| Treprostinil | 6918140 | 390.51 | 86.99 | 4.5 | -4.59 | High | 0 | 0.56 | Antibacterial | Pa>Pi | 0,243 | 0,087 |
| Triamcinolone | 31307 | 394.43 | 115.06 | 1.16 | -2.88 | High | 0 | 0.55 | Antiviral | Pa>0,3 | 0,404 | 0,086 |
| Valsartan | 60846 | 435.52 | 112.07 | 4.39 | -4.97 | High | 0 | 0.56 | Pseudolysin inhibition | Pa>0,3 | 0,387 | 0,179 |
|  |  |  |  |  |  |  |  |  | Muramoyl tetrapeptide carboxypeptidase inhibition | Pa>Pi | 0,256 | 0,179 |
|  |  |  |  |  |  |  |  |  | UDP-N-acetylmuramate-L-alanine ligase inhibition | Pa>Pi | 0,095 | 0,053 |
| Xenazoic acid | 239062 | 375.42 | 75.63 | 4.82 | -5.15 | High | 0 | 0.56 | Antibacterial | Pa>0,3 | 0,354 | 0,042 |
| Zomepirac | 5733 | 291.73 | 59.3 | 2.83 | -3.57 | High | 0 | 0.85 | Cell wall biosynthesis inhibitor | Pa>0,3 | 0,198 | 0,164 |
